# Service user and healthcare-worker perspectives on Substance Use Disorder treatment journeys, relapse, and recovery in Sub-Saharan Africa: Evidence from Uganda

**DOI:** 10.1371/journal.pgph.0005691

**Published:** 2026-08-21

**Authors:** Claire Biribawa, Wouter Vanderplasschen, Joan Nankya Mutyoba, Byamah Brian Mutamba, Kenneth Kalani, Daniel Oyet, David Kalema, Nazarius Mbona Tumwesigye, Vicki Simpson

**Affiliations:** 1 Department of Epidemiology and Biostatistics, School of Public Health, Makerere University, Kampala, Uganda; 2 Department of Special Needs Education, Faculty of Psychology and Educational Sciences, Ghent University, Ghent, Belgium; 3 Butabika National Referral Mental Health Hospital, Kampala, Uganda; 4 Mental Health Division, Uganda Ministry of Health, Kampala, Uganda; 5 School of Nursing, College of Health and Human Sciences, Purdue University, West Lafayette, Indiana, United States of America; PLOS: Public Library of Science, UNITED STATES OF AMERICA

## Abstract

Substance Use Disorder (SUD) treatment in sub-Saharan Africa remains poorly understood from the perspectives of those who deliver and receive it. We integrated the perspectives of service users (SUs) and front-line health care workers (HCWs) within a single analytic frame to map SUD treatment trajectories and identify multilevel barriers and facilitators shaping treatment and recovery outcomes in Uganda. We conducted a qualitative study nested within a parent cohort of adults treated for SUDs at two public treatment facilities in Uganda. We conducted in-depth interviews with 43 SUs from this cohort and 10 HCWs directly involved in SUD care. We analysed the data using reflexive thematic analysis. SU and HCW data accounts were first analysed separately before applying integrative mapping to generate cross-cutting meta-themes. We identified four interrelated meta-themes. First, systemic and structural constraints, including human resource gaps, medication stockouts, and inadequate infrastructure, undermined care consistency and quality. Second, accounts suggested that SUD care was only partially aligned with the biopsychosocial model, with acute biomedical stabilisation more consistently implemented than psychosocial support, individualised care, and recovery planning. Third, participants described returning to high-risk post-treatment environments marked by stigma, weak family support, peer and environmental triggers, economic insecurity, and untreated comorbidities, which increased perceived relapse vulnerability. Fourth, they identified supportive anchors for recovery, including faith and spirituality, active coping, family and community engagement, peer mentorship, task-sharing, and strengthened multidisciplinary care. Participants’ accounts indicate that SUD care and recovery in Uganda are shaped by a self-reinforcing cycle of system constraints, fragmented care, and high-risk post-discharge environments. Reorienting SUD care towards a recovery-oriented continuum will require stronger links between facility-based treatment and community support, alongside investment in psychosocial care, workforce capacity, service infrastructure, and locally available recovery resources.

## Introduction

Substance use disorders (SUDs) remain a persistently neglected driver of global morbidity, mortality, and disability [1–4], with an estimated 400 million people worldwide living with alcohol or drug use disorders [5]. Alcohol use disorders(AUDs) account for the largest share of this burden, while approximately 64 million meet the criteria for drug use disorders (DUDs) [1,4].

The burden of harmful substance use is rising in sub‑Saharan Africa (SSA), where alcohol consumption rates rank among the highest globally, and the prevalence of AUDs varies widely across settings, ranging from 0.1% to 33.2% [6–9]. Alongside heavy alcohol use, the region is contending with an expanding illicit drug landscape encompassing opioids, methamphetamine, and locally produced psychoactive concoctions with severe health and social consequences [10–12]. Despite this burden, access to formal SUD treatment services across SSA remains limited. Most formal treatment services are concentrated in urban areas, and care is fragmented across inpatient and outpatient settings [13]. At the same time, traditional and spiritual healing practices are widespread and often serve as first-line care options, while the vast majority of people who need treatment never receive it [14,15].

Uganda exemplifies the challenges embedded in SSA’s changing substance‑use landscape. The country ranks among the highest globally for per capita alcohol consumption [16], with approximately 10% of the adult population estimated to have an AUD [17,18]. Uganda is also seeing a surge in use of other drugs, including cannabis, codeine, and amphetamine‑type stimulants [19–22]. However, the treatment landscape remains constrained by limited residential facilities, urban concentration of services, gaps in service delivery capacity, continued reliance on traditional healing practices and pervasive stigma that further deters help-seeking [23–25].

Although expanding SUD treatment in SSA is increasingly recognised as a public health priority, less is known about how treatment is experienced and negotiated across the full care trajectory, from facility-based care to community re-entry. Existing studies have tended to examine patient experiences [26–28] and health-system capacity [23,29,30] separately, leaving limited understanding of how service delivery interacts with service user (SU) expectations, treatment engagement, relapse vulnerability, and recovery support. This gap is important because SUD care is shaped not only by the availability of services, but also by how front‑line providers adapt or fail to adapt care within constrained systems to meet patient needs and how SUs navigate these services even after discharge. Integrating these perspectives is essential for understanding mismatches between provider capacity, system readiness and patient expectations [31].

To address this gap, we conducted a qualitative study that integrated the perspectives of health-care workers (HCWs) and SUs within a single analytic frame. We aimed to map the full SUD-care trajectory from admission through community re-entry and to identify multilevel barriers and facilitators shaping treatment experiences, relapse, and recovery support in Uganda.

## Materials and methods

### Ethics statement

Ethical approval was obtained from Makerere University School of Public Health Research Ethics Committee (MakSPH-REC) as part of the AQUALEFF study (Advancing Quality and Effectiveness of Treatment of Substance Use Disorders in Uganda), before data collection. The study was subsequently registered with the Uganda National Council for Science and Technology in accordance with national research guidelines under Registration Number HS6699ES. Additionally, institutional administrative clearance to conduct research at the treatment facilities was obtained from the management of Butabika National Referral Mental Health Hospital and Gulu Regional Referral Hospital, the two public treatment facilities where data collection took place.

All participants provided written informed consent before data collection. Participation was voluntary, and confidentiality and anonymity were assured throughout the study. Data, including transcripts and audio recordings, were securely stored in encrypted digital folders, accessible only to the core research team, to ensure data integrity and confidentiality. Ethical guidelines were strictly adhered to during all stages of the research.

### Study design

This study used a qualitative research design involving in-depth interviews with SUs who had received treatment for SUDs and HCWs directly involved in SUD care. HCWs were interviewed as key informants because of their firsthand experience with treatment pathways, service-delivery constraints, referral processes, and recovery support at the study facilities. The study adopted a constructivist qualitative paradigm [32], which recognises that participants’ accounts of treatment, relapse, and recovery are shaped by their social contexts, lived experiences, and interactions with health services. This paradigm allowed us to examine how SUs and HCWs made meaning of SUD treatment and recovery within their settings. The study is reported in accordance with the 32‑item Consolidated Criteria for Reporting Qualitative Research [33].

### Study setting

The research study was conducted at two public hospitals in Uganda: Butabika National Referral Mental Health Hospital (BNRMH) in Kampala, and the mental-health unit of Gulu Regional Referral Hospital (GRRH) in northern Uganda. BNRMH is the national tertiary centre for mental health and substance-use care. Within BNRMH, the Alcohol and Drug Unit (ADU) functions as Uganda’s only government - run rehabilitation centre for SUDs, offering a full continuum of care. The service pathway begins with medically supervised detoxification; followed by a structured inpatient rehabilitation phase - with a bed capacity of approximately 80 patients involving inpatient care where psychosocial counselling, relapse-prevention planning, group therapy, family involvement and vocational re-integration sessions are provided. Once patients transition from the inpatient phase, the unit offers outpatient follow-up services, comprising scheduled clinics for monitoring, individual and group counselling, peer-support groups and referral back into community-based care or outreach services. The ADU also houses Uganda’s first Medically Assisted Therapy clinic for opioid use disorder, which serves hundreds of clients annually through combined harm-reduction and recovery-oriented care. The staffing mix at BNRMH combines psychiatrists, psychiatric clinical officers, psychiatric nurses, clinical psychologists, counsellors, social workers and peer-support mentors, together forming a multidisciplinary team responsible for assessment, care-planning, treatment implementation and discharge planning.

GRRH serves as the primary mental health regional referral centre for districts in the Acholi sub-region (including the districts of Amuru, Gulu, Kitgum, Lamwo and Pader). GRRH Mental Health Unit is integrated within the general hospital and provides inpatient (approximately 20 beds) and outpatient psychiatric services, managed by psychiatrists and psychiatric clinical officers with linkages to district and community services.

Together, these two settings provided a valuable contrast between national and regional service delivery models. This distinction is important for interpreting differences in service pathways and experiences between the two sites. Interviews were conducted face-to-face in private consultation rooms at the two public facilities.

### Participants, sampling methods and sample size

We included two categories of participants: SU who had received treatment for SUDs, and HCWs directly involved in SUD care at the two study sites. SUs were drawn from a purposively defined parent cohort of 416 adults who had received SUD treatment across two contrasting public health facilities in Uganda: BNRMH (n = 307) and GRRH (n = 109). This parent cohort included individuals aged 18 years or older, with a clinician-confirmed SUD diagnosis according to DSM-5 criteria [34], and documented engagement with treatment services in one of the two study sites. We excluded individuals who presented with psychosis or cognitive impairment that precluded informed consent; clinical teams offered appropriate referrals for these individuals.

From this clinically defined parent cohort, we used a computer-generated random selection procedure to identify SUs for qualitative interviews. Random selection within this bounded cohort was intended to minimise investigator selection bias while preserving heterogeneity across key participant characteristics including sex, age, and substance use patterns. We applied random selection sequentially, selecting participants in batches across both sites, and conducting interim thematic reviews between batches to assess whether substantially new codes or preliminary analytic categories were emerging.

This iterative process continued until thematic sufficiency was reached. Later interviews largely reinforced, elaborated, or provided contextual variation on existing codes and preliminary analytic categories rather than generating substantially new analytic directions. This yielded a final sample of 43 SUs: 25 from BNRMH and 18 from GRRH. Of these, 31 were males and 12 were females, with a mean age of 35 years (range 19– 55 years). The most reported primary substances of use were alcohol (n = 17), cannabis (n = 14), illegal opioids (n = 7), legal opioids such as tramadol and pethidine (n = 4), and sedative-hypnotics (n = 1). Polysubstance use was reported by 20 participants (46.5%).

For HCWs, we employed a purposive sampling strategy to recruit 10 HCWs directly involved in the care of individuals with SUD at the study sites. We treated these interviews as key-informant interviews intended to provide contextual insight into service organisation, treatment pathways, facility-level constraints, referral processes, and perceived barriers to recovery. They included psychiatric clinical officers (n = 3), clinical psychologists (n = 1), psychiatric nurses (n = 2), counsellors (n = 2), and community health workers (CHWs) (n = 2) with firsthand experience in delivering SUD-related services. Their median age was 34 years, and most were male (70%). Psychiatrists, social workers and occupational therapists, present at BNRMH, were not included in the sample; their perspectives represent a gap acknowledged in the limitations.

### Data collection

We collected data between September 2023 and August 2025. This timing reflected the design of the parent cohort study and the need for SUs to have sufficient time to progress through treatment and return to their communities before being interviewed. HCWs were interviewed first to establish a contextual baseline of the service environment, treatment pathways, facility-level constraints, and referral processes at the two study sites. SU interviews were conducted at a minimum of one year after participants’ first engagement with treatment services, to ensure they had accumulated sufficient experience of both facility-based care and post-discharge recovery context, including community reintegration, relapse risks, follow-up challenges, and recovery supports, before being interviewed. Consistent interview domains and data collection procedures were maintained throughout to support comparability across the full collection period.

Interviews were guided by semi-structured interview guides that were tailored separately for SUs and HCWs to capture their distinct experiences and perspectives on SUD treatment, relapse, and recovery. The guides were developed based on the study objectives, literature review, and expert input and included open-ended prompts covering treatment experiences, recovery journeys, pathways to care, healthcare system barriers, and recommendations for improving treatment outcomes. While the guides ensured consistency, interviews remained flexible to accommodate emergent topics and participant narratives. The full interview guides for HCWs and SUs are provided in supporting information file 1 (S1 Text).

The first author, together with trained facility-based research assistants, conducted all interviews in private rooms at the participating facilities to ensure confidentiality. The involvement of facility-based research assistants provided valuable contextual grounding and contributed to the refinement of interview guides and data collection procedures.

Interviews were conducted in English, Luganda, or Acholi, depending on participants’ language preferences. No interpreters were required, as the research assistants were fluent in the respective local languages. Each interview lasted approximately 45–60 minutes and was audio-recorded with participants’ consent. Interviewers also took brief field notes to document contextual observations and non-verbal cues that complemented the transcribed data. All audio recordings were transcribed verbatim and, where necessary, translated into English by the same research assistants which helped preserve meaning and expression of participants’ accounts. Transcripts were reviewed for completeness and accuracy before analysis.

### Data analysis

We analysed the data using Braun and Clarke’s six-step framework for reflexive thematic analysis [35]. This approach aligned with our constructivist paradigm and enabled us to identify and interpret patterns of meaning across participant narratives while remaining attentive to individual and contextual factors influencing treatment journeys and outcomes.

Two independent analysts with complementary backgrounds read, re-read and coded the transcripts to gain a comprehensive understanding of the content and record preliminary impressions. The first author (CB), who has a background in public health and professional experience in SUD research in Uganda, brought theoretical sensitivity and contextual knowledge of Uganda’s SUD treatment landscape. The second analyst (VS), who has a background in psychology and qualitative research and is based outside the SSA context, brought an external analytic perspective. This combination allowed us to balance contextual familiarity with critical distance during analysis.

CB and VS analysed interviews from SUs and HCWs separately in the initial stages to preserve group-specific meanings and contexts. We then developed initial codes manually through close reading of the transcripts, highlighting meaningful text segments related to treatment experiences, relapse, recovery, health-system constraints and sources of support.

The analysts met regularly to compare coding decisions, review codes against extracts, discuss emerging interpretations, and refine the coding framework. Coding consistency was ensured through transparency of the analytic process, systematic documentation of analytic decisions and reflections, and all interpretive differences were resolved through discussion and reference back to the raw data.

We then clustered related codes into sub-themes, which represented closely connected ideas or processes within each participant group’s accounts. After developing sub-themes separately for SUs and HCWs, we conducted a comparative analysis to examine how these sub-themes aligned, overlapped, or contrasted across the two datasets. Through iterative team discussions and integrative mapping, we synthesised these findings into a set of cross-cutting meta-themes that captured shared experiences as well as group-specific insights on treatment, relapse, and recovery. We assigned clear definitions and labels to each sub-theme and meta-theme to ensure they accurately reflected the underlying data and participant narratives. The broader research team also reviewed emerging themes and discussed alternative interpretations.

We assessed thematic sufficiency through interim analysis alongside data collection and final analysis after data collection was complete. During interim analysis, the research team reviewed transcript and emerging codes after each batch of SU interviews to determine whether additional interviews were generating substantially new codes. As coding progressed, later SU interviews largely reinforced, elaborated, or provided contextual variation on existing codes and preliminary categories rather than generating new major analytic directions. For HCWs, thematic sufficiency was assessed in relation to their role as key informants rather than as a representative sample. The 10 HCWs were purposively selected because they had direct experience of SUD service delivery at the two study sites and could provide information on treatment pathways, facility-level constraints, referral processes, and perceived barriers to recovery. During analysis, HCW accounts showed consistency around main system-level and service-delivery issues.

### Trustworthiness/credibility

We enhanced trustworthiness of the study findings through triangulation across SU and HCW accounts, independent coding of transcripts by two analysts, regular analytic discussions, documentation of coding decisions, and systematic comparison of convergent and divergent perspectives. In addition, emerging themes and illustrative quotations were reviewed by co-authors (DO and KK) with direct facility-based experience in SUD care in Uganda to assess interpretive accuracy, contextual resonance, and plausibility. This process strengthened the credibility, dependability, and analytic coherence of the findings.

### Inclusivity in global research

Additional information regarding the ethical, cultural, and scientific considerations specific to inclusivity in global research is included in the S1 Checklist.

## Results

We present findings from in-depth interviews with 43 SUs who had received treatment for SUDs and 10 HCWs directly involved in SUD care in two public treatment facilities in Uganda. Four interrelated meta-themes traced the SUD-care trajectory from facility-based treatment through discharge, community reintegration, relapse vulnerability, and recovery support. The first three meta-themes primarily capture barriers and vulnerabilities across this continuum, while the fourth meta-theme captures facilitators of recovery, that may reduce relapse vulnerability and function as supportive anchors for sustained recovery (Table 1).

**Table 1:** Overview of meta-themes and sub-themes derived from service user and healthcare worker perspectives on SUD treatment and recovery in Uganda.

Meta-theme	Sub-themes
Systemic and structural constraints in SUD treatment	• Human resource constraints • Medication and supply shortages • Security gaps and in-facility substance access • Infrastructural constraints • Inadequate basic support systems and facility conditions • Geographic inequities in SUD care access
Fragmented implementation of comprehensive SUD treatment	• Partial implementation of the biopsychosocial model • Limited individualisation and patient involvement • Insufficient inpatient duration and weak continuity of care • Unmanaged side effects and medication-related concerns
Post-treatment vulnerabilities and recovery Challenges	• Gaps in continuing care and reintegration into community • Stigma and moral labeling • Inconsistent family support • Environmental, peer, and psychological triggers • Comorbid mental and physical health challenges
Pathways and supportive anchors for sustained recovery: facilitators of recovery	• Faith, spirituality, and search for meaning • Active coping and lifestyle change • Family and community engagement • Peer-led recovery • Systems strengthening and multidisciplinary care • Task-sharing to strengthen community-based care continuity

The four meta-themes were analytically interconnected rather than discrete categories. Together, they described a treatment and recovery pathway in which structural constraints shaped the form and quality of facility-based care, fragmented implementation of comprehensive SUD treatment limited preparation for community re-entry, and post-discharge social environments increased vulnerability to relapse. SUs and HCWs often described the same system from different positions: HCWs highlighted service-delivery constraints, while SUs described how these constraints affected trust, participation in care, treatment continuity, and recovery after discharge. Although site-specific differences were also evident across the two facilities, shared themes pointed to broader structural weaknesses in SUD care.

### Meta-theme 1: Systemic and structural constraints in SUD treatment

This meta-theme described how shortages of staff, medicines, infrastructure, security, and basic patient support shaped the conditions under which SUD care was delivered. HCWs experienced these constraints as limits on what they could provide, while SUs experienced them as inconsistent, incomplete, or inaccessible care. However, the form of scarcity differed by site: BNRMH accounts centred on a highly utilised national referral unit where available resources were insufficient to meet patient volumes and specialist demands, while GRRH accounts highlighted the vulnerability of a regional service operating with limited service delivery capacity.

#### Sub-theme 1.1: Human resource constraints.

Human resource shortages operated as a central mechanism through which system-level constraints became clinical constraints. At both study sites, participants described limited specialist staff, high patient-to-provider ratios, and role substitution which led to work overload, reduced time for comprehensive assessment, counselling, individualised care planning, and follow-up. HCWs framed these shortages not simply as workload problems, but as barriers to delivering the kind of holistic care they believed SUD patients required.

*“Human Resource is one of the major challenges we face…there is no attached counsellor or psychologist at the unit, we try to fill up these gaps but haphazardly.” (HCW #3)*

These staffing pressures deepened providers’ sense of helplessness and contributed to moral distress from a recognition of what holistic, patient-centred treatment should entail alongside a structural inability to deliver it. Beyond workload, poor remuneration and slow career progression further eroded staff motivation, threatening workforce retention.

*“Someone can take even 10 years to be promoted… and yet you have the qualifications. So, there’s the way it demoralises even the people who are working.” (HCW#6)*

#### Subtheme 1.2: Medication and supply shortages.

Medication shortages revealed how a single system constraint could produce different meaning across participant groups. HCWs described shortages as limiting their ability to provide comprehensive pharmacological care, often forcing them to rely on psychosocial approaches or symptomatic management. SUs experienced medicine stockouts as inconsistent, incomplete, and inequitable care, especially when they were asked to purchase medicines privately.

*“When the patient cannot afford to buy some of these medications… we divert to psychological measures such as distraction, exercise, sleep medication.” (HCW#5)*

*“They tell you to go buy this medicine because it is not there at the hospital, you reach the pharmacy, and it is very expensive.” (SU#11)*

#### Sub-theme 1.3: Security gaps and in-facility substance access.

Security vulnerabilities represented a distinct form of structural failure, one in which the treatment environment itself became a site of continued substance use, directly undermining treatment. The nature of this vulnerability differed by site, reflecting each facility's physical design and security capacity. HCWs at BNRMH reported inadequate security screening procedures, which facilitated smuggling of psychoactive substances into inpatient wards.

*“We don’t have a good security… drugs sneak in…so you are trying to treat the patient… when you do a drug screen you find he used drugs while still on the ward.”* (HCW#4)

At GRRH, the absence of a perimeter wall allowed patients to move out freely to access substances or engage with community members using substances.

*“Our unit is an open space, and patients come in and go out whenever they want. Substances sneak in, so you are detoxing, but the patient is taking substances. (HCW#2)*

#### Sub-theme 1.4: Infrastructural Constraints.

Across both facilities, participants described overcrowding, inadequate bed space, and lack of private areas for counselling. In both facilities, patient numbers frequently exceeded available capacity, resulting in the placement of some individuals in acute psychiatric wards that were not designed for addiction rehabilitation.

*“When facilities become overwhelmed by bed capacity, patients are discharged at the first sign of slight improvement, even if they aren’t clinically ready.” (HCW#2)*

#### Sub-theme 1.5: Deficits in essential patient support and facility living conditions.

Unmet basic needs, particularly food insecurity and poor sanitation, emerged as a GRRH-specific finding that illustrated how structural neglect can undermine treatment retention before any clinical intervention takes effect. GRRH operated without systematic food provision for inpatients, creating a situation in which patients faced a choice between staying in treatment and meeting their basic nutritional needs.

HCWs highlighted that some inpatients were discharged prematurely or absconded because they could not get meals during their stay. Patients who left the ward to seek food frequently returned re-intoxicated or failed to return at all.

*“Patients aren’t provided with food so they are discharged prematurely because they cannot afford to be here without food, or they just run away.” (HCW#3)*

#### Sub-theme 1.6 Geographic inequities in SUD care access.

Geographic inequity emerged as a structural barrier that shaped both entry into care and continuity after discharge. Both HCWs and SUs highlighted that publicly funded SUD services were highly centralised, forcing patients from rural and distant districts to bypass lower-level facilities and travel directly to regional or national referral hospitals. HCWs interpreted this as a weakness in the referral system and in the capacity of lower-level health facilities to identify, manage, or follow up SUD cases. SUs experienced the same problem as delayed access, high transport costs, and difficulty sustaining follow-up after discharge.

*“The health system levels from general hospital right to Health Centre II are not helping. People bypass all those levels and come to the regional referral.” (HCW #1)*

### Meta-theme 2: Fragmented implementation of comprehensive SUD treatment

This meta-theme showed how structural constraints described in Meta-theme 1 translated into fragmented SUD care across treatment facilities. Despite Uganda’s treatment framework being conceptually grounded in a biopsychosocial model, participants’ accounts suggested that biomedical stabilisation was the most consistently delivered part of care while psychosocial, relational, and rehabilitative components were more difficult to implement consistently. This was reflected in limited patient engagement in treatment planning, brief inpatient admissions that constrained recovery consolidation, and inadequate management of medication-related side effects. This gap between the intended model and the delivered model was one of the cross-cutting patterns across both sites and participant groups.

#### Sub-theme 2.1: Partial implementation of the Biopsychosocial model.

HCWs at BNRMH and GRRH acknowledged the biopsychosocial model as the intended framework, but participants’ accounts suggested that care often leaned towards biomedical stabilisation, particularly detoxification, acute symptom management, and management of co-occurring mental health symptoms. Psychological and social components, including counselling, occupational therapy, and family engagement, were inconsistently implemented because of staffing shortages and logistical challenges. Providers described a system delivering less than intended, while for SUs it was less than they needed. These perspectives reveal biomedical dominance not as a clinical choice but as a default outcome of structural under-resourcing

*“When I came here, I was not encouraged to stop using substances, I did not get any counselling…. I thought I was just forced to stop taking marijuana.” (SU#9)*

#### Sub-theme 2.2: Limited individualisation and patient involvement in treatment.

Accounts from both HCWs and SUs highlighted a lack of individualised care and patient participation in the treatment process. Participants described SUD care as largely standardised, with limited collaboration and adaptation to individual needs. High caseloads limited time for comprehensive assessments and developing personalised treatment plans.

*“…we do not do thorough assessments to critically understand the categories of our patients. Some of our patients are brought to the unit tied but we fail to classify them and treat them in the one size fits all way.” (HCW#3)*

From the SU perspective, the absence of individualisation extended beyond assessment into the therapeutic relationship itself. SUs described receiving medication without adequate explanation, entering treatment under family pressure, and being given little opportunity to participate in decisions about their own care which undermined therapeutic alliance and treatment engagement.

*“I was forced to take medication which I do not agree with and instead of it helping me, it just hindered me because I do not agree with taking pills when I am not sick.” (SU#6)*

#### Sub-theme 2.3: Insufficient in-patient duration and transition in care.

Across facilities, HCWs reported that inpatient treatment durations were shorter than they considered adequate for sustained recovery. At BNRMH, they noted stays averaged at 2–2.5 months but were often truncated due to high patient turnover and limited bed capacity. At GRRH, HCWs described typical admissions of about two weeks, acknowledging that such brief stays were insufficient for meaningful behavioural and psychosocial stabilisation.

*“… we tend to have a huge number of patients waiting to be admitted to the ADU and sometimes when you feel the patient has been able to get this and that, we just continue with him as an outpatient to create some space.” (HCW#9)*

#### Sub-theme 2.4: Unmanaged side-effects and medicine dependence.

Medication-related side effects become problematic for SUs when they were not accompanied by adequate explanation, monitoring and management. SUs reported distressing side-effects, including sedation, drowsiness, sexual dysfunction, physical discomfort, and disturbed sleep, often without adequate discussion with clinicians. For some participants, these experiences generated confusion, fear, and reduced trust in treatment. Some questioned whether treatment itself was harmful and expressed anxiety about developing dependence on prescribed medications.

*“I feel addicted to these tablets, because medication is something that replaced drinking. You feel like you are a slave to this medication.” (SU#12)*

This account illustrates a therapeutic paradox where treatment was perceived by SUs as substituting one form of dependence for another. Without adequate explanation of how prescribed medications work, why they are used, and how they differ from substances of misuse, some SUs interpreted their treatment experiences through the lens of their prior relationship with substances. This reflected the broader pattern of limited patient involvement and therapeutic dialogue described in Sub-theme 2.2. In some accounts, unmanaged side effects appeared to increase vulnerability, as SUs turned to familiar substances to manage distressing symptoms that they felt treatment had failed to address.

*“…… the hallucinations continued coming, and I decided to take alcohol to help me deal with them, which spoilt my sobriety, and I was taken back ….” (SU#4)*

### Meta-theme 3: “Life beyond the ward”: Post-treatment vulnerabilities and recovery challenges

This meta-theme captured how recovery extended beyond the hospital setting and unfolded within social environments that could either support or undermine change. The post-discharge period emerged as key point of vulnerability in the SUD continuum, where the service system constraints described in Meta-themes 1 and 2 intersected with SUs’ everyday realities. For many SUs, community re-entry meant returning to environments and relationships that had previously enabled substance use, including contexts marked by weak continuing care, stigma, family strain, peer influence, poverty, substance availability, and untreated comorbidities. HCWs tended to describe these vulnerabilities as gaps in the care continuum, while SUs described them as everyday pressures that reactivated cravings and undermined motivation after discharge.

#### Sub-theme 3.1: Gaps in continuing care and community reintegration.

Continuing care after discharge emerged as the most structurally absent component of the SUD care continuum. At BNRMH, HCWs described a clear intention to continue care after discharge through reviews, family sessions, peer/community arms, and even home visits but were rarely implemented due to the same resource constraints described in Meta-theme 1.

*“In our treatment plan, we’re even supposed to do home visits… but with limited staff and many patients… we are unable to do this.” (HCW#4)*

Community-level support peer structures such as the Uganda Harm Reduction Network and AA/NA-type groups were reported to exist but reached only a small proportion of discharged SUs, primarily in Kampala, leaving those in peri-urban and rural areas with no community-level recovery infrastructure. Similar challenges were reported at GRRH, where HCWs cited the absence of relapse prevention strategies to provide ongoing support. HCWs described a passive, reactive system in which follow-up was contingent on patients returning voluntarily rather than being proactively supported.

*“There is no proactive follow-up of the patients in the communities to understand how they manage. We just sit back and wait, hoping they come back for review.” (HCW #2)*

#### Sub-theme 3.2: Stigma and moral labelling.

Both HCWs and SUs reported how moralistic perceptions, stigmatising language, and labelling undermined SUD recovery. Stigma arose from various sources including HCWs, families, and communities. SUD was often viewed as a moral failing or sign of madness, leaving participants feeling rejected, misunderstood, and ashamed.

*“When I went back home, people still called me names, mad, addict… because they thought I was still using.” (SU#17)*

#### Sub-theme 3.3: Inconsistent family support in SUD care.

Family involvement emerged as both a facilitator and a source of vulnerability in SUD recovery, depending on the nature and quality of family engagement. Some SUs reported that their families offered emotional, logistical, or financial support which motivated recovery.

*“What I can say is my family members, they supported me a lot. When I relapsed, they rushed me to the hospital…when I don’t have money, they buy medicine for me.” (SU#13)*

However, supportive families were the exception. Some participants described relatives as emotionally exhausted, financially strained, judgmental, or absent after repeated episodes of relapse and treatment. In other cases, family conflict, poor communication, stigma, or substance use within the household created stressors that undermined recovery after discharge. Thus, family involvement functioned less as a uniformly protective factor and more as a relational resource whose effect depended on whether families were supportive and able to participate constructively in care.

*“...by the time the person comes to the facility they are treated as rejects and the relatives have been pushed to the limits, they rarely support.” (HCW #2)*

Even well-intentioned families sometimes contributed to relapse through poor communication, unresolved trauma, and emotionally charged encounters. In these cases, family was not merely absent as a protective factor but present as a relapse trigger.

*“When you have people who are generally judgmental, then I tell myself maybe let me have a beer first. You reach home, and your wife starts shouting....” (SU#4)*

#### Sub-theme 3.4: Environmental and peer-driven triggers.

Both HCWs and SUs indicated that recovery was challenged by peer pressure and environments saturated with alcohol and drug use. Respondents consistently reported that the moment they left the ward, they were “thrown back into a sea of substances.” Some participants explained that local brewing is a source of livelihood, and this makes recovery hard.

*“I have a patient who is always coming back to the facility with alcohol induced psychosis…her employment is brewing local alcohol... She usually tells me that you cannot sell what you have not tasted.” (HCW#9)*

Many SUs described being drawn back into familiar social networks where substance use remained normalised. Peers of SUs dismissed HCW advice and offered substances as gestures of friendship, reactivating cravings and undermining progress made. This revealed how community social norms can directly compete with and undermine clinical guidance.

*“My friends refuted HCWs’ statements against my drinking abstinence…saying they are just foolish and that alcohol does nothing to a person…” (SU#24)*

#### Sub-theme 3.5: Social and psychological triggers.

SUs described relapse vulnerability as closely tied to everyday social and psychological pressures, including social isolation, emotional distress, and economic insecurity with limited follow-up. These triggers were interconnected to other vulnerabilities: stigma eroded social connectedness, family strain removed emotional support, poverty generated chronic stress, and the absence of continuing care left SUs to navigate these pressures without clinical guidance. Some participants described substance use as a way of coping with loneliness, frustration, financial hardship, or despair. In extreme cases, this psychological burden extended to suicidality, with substance use intertwined with attempts to escape from despair.

*“I had come back from prison, with loads of thoughts and felt alone…I went to the bush with poison thinking that I would have my last marijuana then drink the poison, but after smoking marijuana, I felt life became meaningful again.” (SU#32)*

#### Sub-theme 3.6: Comorbid mental and physical health challenges.

Comorbid mental and physical health conditions added another layer of complexity to SUD treatment and recovery. Both HCWs and SUs reported that co-occurring mental health comorbidities such as depression, anxiety, and bipolar disorder complicated treatment and undermined recovery. Participants also described physical health problems that increased stress and affected daily functioning. These accounts highlighted the difficulty of addressing SUD in isolation from broader mental and physical health needs. A related concern was that some SUs with polysubstance reported receiving treatment of one primary substance while continuing to use several others concurrently. This suggested that incomplete clinical assessment of comorbidity and polysubstance use could leave recovery needs insufficiently addressed.

*“…Even when you run my blood sugar levels right now, they are high…then I get episodes of hypoglycemia, I start sweating, and people start laughing at me that that is alcohol! Stupid, I am dying here, and you are busy telling me that?” (SU#4)*

#### Meta-theme 4: Pathways and supportive anchors for sustained recovery.

This meta-theme describes the facilitators of recovery that participants identified across individual, interpersonal, community, and health-system levels. Unlike preceding meta-themes, which described constraints and vulnerabilities that increased relapse risk, this theme highlights resources that could interrupt the relapse cycle and support recovery beyond the facility. SUs described spirituality, parental responsibility, structured routines, and supportive relationships as personal and relational anchors for sobriety. HCWs emphasised peer mentors, family engagement, task-sharing, multidisciplinary care, and stronger referral systems as potential ways to extend recovery support.

#### Sub-theme 4.1: Faith, spirituality and search for meaning in recovery.

Faith and spirituality were described as important forms of personal and social recovery capital. SUs described faith, spiritual belief, prayer, and religious community engagement as sources of internal motivation, discipline, hope, and accountability. Both Christian and Muslim participants described religion as a stabilising influence that helped them resist peer pressure, reframe their purpose, and provided a sense of belonging and frameworks for daily discipline, self-reflection, and community accountability.

*“I listen to Christian radio called ‘Favour FM’, it really encourages me not to take alcohol” (SU#30)*

For some, faith communities also provided practical roles and supportive relationships that reduced idleness and social isolation. Participants viewed religious leaders as accessible community-based sources of encouragement who could complement formal treatment, especially when distance, cost, or limited follow-up made hospital-based counselling difficult to sustain. In this sense, spirituality functioned not only as an individual coping resource but also as a potential bridge between facility-based care and community recovery support.

*“Currently I am managing the church public address system, and this keeps me busy, and I don’t have free time to use drugs… In case of challenges, I share with my pastor, and I get words of encouragement and advice.” (SU#7)*

Parental responsibility also emerged as an important source of meaning and motivation. Some SUs described prioritising their children’s basic needs as a reason to reduce spending on substances and remain focused on recovery.

*“These days, when I get some money, I first think about getting food for my children before anything else.” (SU#30)*

#### Sub-theme 4.2: Active coping and lifestyle change.

SUs described active coping and lifestyle change as self-directed resources for maintaining recovery after discharge. They framed recovery as requiring the deliberate replacement of substance-related routines with structured, meaningful, and physically engaging activities. Farming, exercise, household responsibilities, and other purposeful daily tasks helped reduce idle time, manage cravings, and create distance from previous substance-use networks.

*“What is helping me now is I am doing farming these days…. it keeps me busy that I don’t have free time to spend, I come back from the garden when I am tired.” (SU#26)*

*“I go running in the morning and in the evening…if you were thinking about taking opium you will not... you just keep yourself busy.” (SU#15)*

These accounts suggest that recovery support should not focus only on clinical stabilisation, but also on helping service users develop realistic post-discharge routines, livelihood options, and coping strategies that can be sustained within their everyday environments.

#### Sub-theme 4.3: Community and family engagement for SUD recovery.

Both HCWs and SUs highlighted family and community involvement as important for supporting recovery beyond the hospital. Participants proposed family counselling, community sensitisation, and engagement of religious and local leaders to reduce stigma and strengthen community reintegration.

*“We need to conduct sensitisation of the community about addictions, and this should start from schools.” (HCW#8)*

SUs also urged facilities to incorporate structured family counselling. They believed that when relatives understand addiction and receive guidance alongside the patient, they become allies in monitoring stressors and preventing relapse.

*“…I feel the medical team should provide more education to the care takers of the patients who come to hospital to get treatment.” (SU#9)*

#### Sub-theme 4.4: Peer-led recovery; role of expert clients.

In addition to formal treatment systems, both HCWs and SUs valued peers with lived experience of recovery, sometimes referred to as “expert clients”, as credible sources of support. Participants described expert clients as people who could relate to SUs’ struggles, model the possibility of change, and offer practical guidance after discharge. Unlike formal providers, expert clients were seen as able to communicate from shared experience, making their advice more acceptable and trustworthy. HCWs also envisioned structured roles for them within the continuum of care, including training and empowering them to support the recovery of others.

*“Since we’re complaining of human resources, we can use our very patients who are really complying with treatment to be the mentors in their communities… it gives them purpose and helps prevent relapse.” (HCW #10)*

This finding suggests that peer-led models could strengthen continuity of care by linking clinical treatment to community-based recovery support.

#### Sub-theme 4.5: Strengthening infrastructure and multidisciplinary care systems.

HCWs described infrastructure strengthening and multidisciplinary care as necessary conditions for extending SUD treatment beyond acute stabilisation. Infrastructure needs included safer ward environments, controlled access to treatment spaces, and facility improvements that could reduce in-facility substance access and support more structured care. Participants also emphasised the need to strengthen technical capacity through staff recruitment, training, and functional referral networks. These system-level improvements were viewed as important for delivering more comprehensive care that integrates biomedical, psychosocial, rehabilitative, and community-based support.

*“That's why on the referral system it should be both external referral from outside the hospital, from the community, then also from within the hospital, which is an interdepartmental referral system for a multidisciplinary approach.” (HCW#5)*

#### Subtheme 4.6: Task sharing to strengthen community-based care continuity.

In response to critical human resource shortages and fragmented follow-up systems, HCWs proposed task-sharing as a way to extend SUD care beyond hospital settings. They linked weak follow-up partly to shortages of specialist staff, but also argued that existing community-level actors, particularly Village Health Teams (VHTs) and lower-level health workers, could support recovery if appropriately trained, supervised, and motivated. They emphasised the pivotal role of VHTs, who are the first point of contact within the health system and are well positioned to provide basic psychosocial support, monitor progress after discharge, and strengthen referral linkages between communities and health facilities.

*“We often claim that we don’t have enough human resources or funding, but that’s not entirely true; we have people at the grassroots level, VHTs, get them to do trainings, motivate them, give them additional things, they can do this work very well.” (HCW #10)*

This finding positions task-sharing as more than a workforce solution. It represents a potential mechanism for improving continuity of care, decentralising recovery support, and reducing dependence on overstretched referral facilities.

### Integrative thematic framework: Linking barriers, vulnerabilities, and pathways to SUD recovery

**Fig 1:**
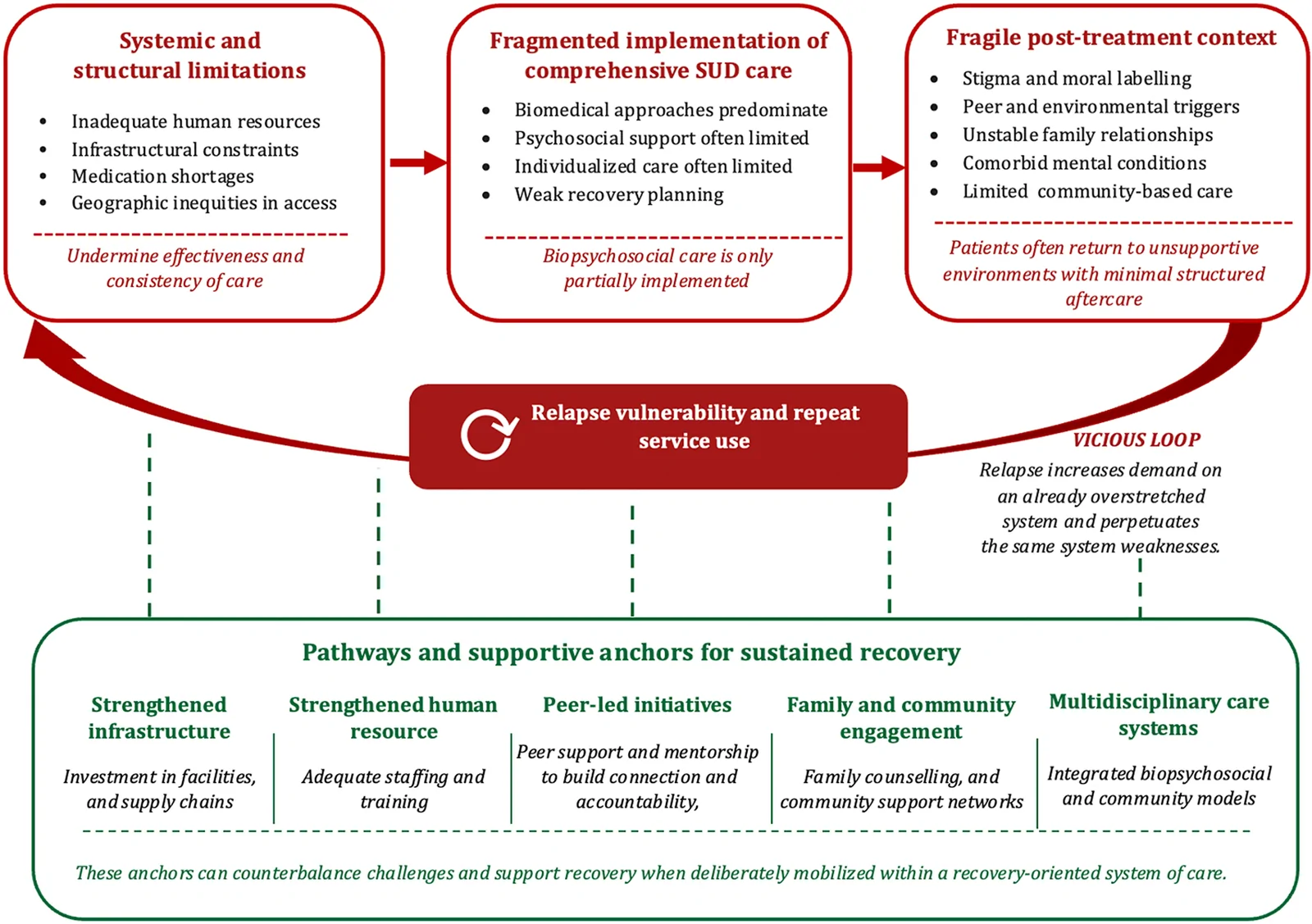
Self-reinforcing cycle of systemic and social dynamics of relapse and recovery in SUD care in Uganda.

Based on the four meta-themes, Fig 1 presents an integrative thematic framework showing how barriers, vulnerabilities, and recovery-support resources interact across the SUD-care continuum. The red pathway illustrates how systemic and structural constraints, fragmented implementation of comprehensive SUD care, and fragile post-treatment environments were interconnected and perceived to jointly shape relapse vulnerability and repeat service use. The vicious loop shows how relapse and repeat service use may increase pressure on an already overstretched system, thereby reinforcing the same service gaps. The green pathway illustrates the supportive anchors identified by participants, including strengthened infrastructure, enhanced human-resource capacity, faith and spirituality, active lifestyle coping, peer-led initiatives, family and community engagement, and multidisciplinary care systems. Together, the framework shows how recovery-support resources may help interrupt this cycle when deliberately mobilised within a recovery-oriented system of care.

## Discussion

This study integrated the perspectives of SUs and HCWs to examine the SUD-care trajectory in Uganda, from facility-based treatment through discharge and community reintegration. By bringing these perspectives into a single analytic frame, the study extends limited SSA evidence on how health-system capacity, service delivery, and social context interact to shape SUD treatment and recovery. It also shows how mismatches between provider constraints and SU needs may undermine treatment engagement and recovery support, even where providers are delivering the best care possible within resource-constrained settings.

Findings showed that SUD treatment and recovery were shaped by a dynamic interaction between health-system constraints, service-delivery practices, post-discharge social environments, and individual factors. These influences did not operate as isolated barriers but formed a self-reinforcing cycle across the treatment and recovery continuum. Structural and resource constraints limited the delivery of comprehensive SUD care, resulting in care that participants perceived as more consistently oriented towards the biomedical model, which prioritised detoxification and acute stabilisation over sustained recovery support, including counselling, occupational therapy, relapse prevention planning, and continuing care. Within this model, many SUs described returning to environments that had previously supported their substance use, including contexts marked by stigma, poverty, strained family relationships, peer influence, and substance availability. Importantly, they often returned without adequate preparation to recognise relapse triggers or adopt behavioural strategies to maintain abstinence. Similar patterns have been reported in other LMICs, where biomedical dominance persists [36,37], and rarely progresses to psychosocial or community rehabilitation despite policies promoting holistic care [38–40]. This mismatch was perceived by participants to heighten relapse vulnerability and contribute to repeat service use, further straining an already over-stretched system [41].

Closely linked to this imbalance was a lack of individualised and participatory treatment planning. Participants described standardised “one-size-fits-all” approaches that left little room for person-centred assessment, collaborative goal setting or discussion of the specific risks each SU would face after discharge. Such limited personalisation may fail to capture the heterogeneity of substance-use trajectories, including differences in clinical complexity, psychosocial stressors, family support, substance-use patterns, and post-discharge recovery environments. This has implications for treatment engagement, adherence, therapeutic alliance, and preparation for community re-entry [13,42–44]. In contrast, person-centred models have been associated with better patient-reported recovery outcomes [45].

These gaps become clearer when interpreted through a recovery-oriented systems of care (ROSC) lens. ROSC emphasises that recovery is not limited to detoxification or symptom stabilisation, but requires coordinated supports across treatment entry, recovery initiation, recovery maintenance, and community life [46]. However, the findings suggested that Uganda’s public SUD services provided some support at the stabilisation stage but were weak at the stages of recovery planning, relapse-prevention preparation, continuing care, and community reintegration. Similarly, the Chronic Care Model argues that long-term conditions require coordinated, community-linked, patient-centred care rather than episodic acute intervention [47]. Together, these frameworks help explain why a model focused mainly on acute stabilisation may be insufficient for SUs returning to environments where substance use remains socially, economically, or emotionally embedded. However, participants did not describe relapse as inevitable. At multiple points in the treatment and recovery continuum, they identified supportive anchors that could disrupt this cycle. These accounts also highlighted recovery-support resources that could be mobilised beyond the facility.

One central finding was the shortage of resources and infrastructure across SUD treatment facilities. These constraints shaped care quality, continuity, and recovery support, and mirror national readiness assessments [23] and broader patterns observed across SSA [13,26,48–52]. However, these constraints differed by setting. BNRMH, as a national referral facility, faced pressures linked to high patient volumes, bed turnover, and specialist service demand. GRRH, as a regional referral hospital, reflected a different set of constraints, including limited specialised SUD services, weaker infrastructure, an open unit layout that made substance access difficult to control, and long travel distances that complicated access and follow-up for rural patients. These differences suggest that implementation strategies should be tailored to facility level and context. National referral facilities such as BNRMH may require investment in strengthening specialist multidisciplinary teams, structured discharge planning, reliable access to essential SUD medicines, and infrastructure that can support high patient volumes. Regional level priorities may include decentralisation of basic SUD care, developing referral and community follow-up mechanisms, and task-sharing with trained non-specialist providers. Across both levels, stronger linkages with lower-level facilities would be important for improving continuity of care beyond referral hospitals.

Medication shortages represented a particularly consequential dimension of these resource constraints. The reported limited availability of essential psychotropic and anti-craving medications such as naltrexone and buprenorphine mirrors broader patterns observed across LMICs, where frequent stockouts, weak logistics systems, and constrained budgets disrupt the management of chronic mental health conditions [41,53–55]. In the absence of medicines, clinicians relied on psychosocial approaches and symptomatic management. Although these approaches are important components of SUD care, relying on them as substitutes for unavailable medicines limited clinicians’ ability to provide comprehensive and individualised treatment [56]. Medication unavailability was perceived by participants to contribute to disengagement from care. The practice of asking patients to purchase medications privately, though intended to bridge supply gaps, increased inequities by excluding those who could not afford them. Addressing these deficits requires strengthening procurement, forecasting, financing, and supply-chain systems to ensure consistent availability of these medicines at public treatment facilities.

Resource constraints also shaped the human-resource and follow-up gaps identified by participants. To address critical workforce shortages and fragmented follow-up systems, HCWs recommended task-sharing with CHWs and other non-specialist providers. This aligns with global mental health literature, which supports task-sharing as a practical and scalable strategy for LMICs [57,58]. Recent reviews and empirical studies have shown that non-specialist interventions incorporating motivational interviewing, psychoeducation, and problem-solving techniques were effective across LMICs [59–61]. Importantly, participants did not frame task-sharing merely as a generic workforce solution but identified CHWs as trusted and accessible figures already embedded within the communities and social environments to which SUs return after discharge. In this sense, task-sharing may function not only as a human-resource strategy, but also as a mechanism for community reintegration and continuity of care. Uganda’s previous experience with CHW-led HIV and mental-health programs further supports the feasibility of this model [62].

However, task-sharing is not self-sustaining, and without sustained supervision, motivation, and clear role boundaries, lay health worker programs may experience high attrition, role overload, and diminishing fidelity over time, risks that are particularly salient in Uganda's already strained health system [58]. For task-sharing to strengthen SUD care without overburdening CHWs, it would require structured training in SUD-specific psychosocial support, defined referral pathways, and integration within existing community health structures.

Participants also highlighted the role of ‘expert clients’ or peers with lived experience of recovery who could mentor others and bridge clinical and community-based recovery support. Peer-facilitated recovery groups have demonstrated acceptability and improved functional outcomes, suggesting their potential applicability for SUD care [63–65]. Integrating structured peer-support systems within Uganda’s SUD care continuum could strengthen continuity between inpatient and community care and increase recovery capital. However, such models would also require training, supervision, safeguards, and referral linkages to avoid placing unsupported responsibility on people in recovery.

Family support emerged as both a facilitator and barrier to recovery, a duality that single-perspective studies may flatten into either a positive or negative. Participants described families as critical sources of emotional stability, practical assistance, and motivation during treatment, findings consistent with prior research [66–69]. However, for many, families were either absent, strained, or emotionally exhausted by repeated relapses and financial burdens of care [70]. In some cases, family environments were themselves sources of trauma, abuse, stigma, or substance exposure and have been linked to poor recovery outcomes [71,72]. This contextual heterogeneity suggests that family engagement in Uganda's SUD care system should be needs-assessed rather than universally prescribed, distinguishing between families who need psychoeducation and support to become recovery allies [73] and those whose involvement requires careful clinical management.

Importantly, stigma was not confined to the community but also originated within treatment facilities, where moral stigma, stigmatising language, and negative stereotypes shaped both treatment experiences and community reintegration. Labelling individuals as “addicts” or “abusers” can reinforce self-stigma and deter individuals from accepting or continuing treatment [74–77]. Similarly, fear of being judged or negatively labelled by society may encourage secrecy and reluctance to disclose struggles with SUD. This suggests that SUD care in Uganda should include stigma-reduction interventions at both facility and community levels, including HCW training on non-stigmatising language, alongside community sensitisation that frames SUD as a health condition rather than a moral failing.

Several post-discharge vulnerabilities reflected the wider social and economic conditions in which recovery unfolded. A notable finding from this study was the economic entanglement between substance use and livelihood, where alcohol brewing or vending provided a primary income source for some participants, as reported in other African contexts [78,79]. This suggests that recovery support may need to address livelihood alternatives alongside clinical care. Evidence from Kenya suggests that livelihood-replacement programs can reduce hazardous drinking [78,80].

Peer networks also shaped recovery trajectories. Both SUs and HCWs described peers as powerful drivers of relapse, consistent with social-learning theory, which indicates that behaviour is shaped and maintained through observation, reinforcement, and social belonging [81]. Some studies have shown that social interactions, particularly with peers who engage in substance use, can increase relapse following treatment [82–84]. Integrating peer-refusal skills and social network planning into routine counselling may improve post-discharge recovery preparation, as reported in some studies [85–87].

Alongside these risks, participants identified locally meaningful recovery resources. Faith-based coping also emerged as an important source of meaning, accountability, social belonging, and motivation. These accounts are consistent with Ugandan evidence linking religiosity to reduced harmful alcohol use [88,89]. However, faith-based support should not be viewed as a substitute for clinical care. Rather, when appropriate and acceptable to SUs, faith leaders and religious communities may provide locally accessible recovery support that complements formal treatment and community follow-up.

Similarly, participants described poverty, unemployment, and idleness as recurring stressors, while purposeful activity emerged as a practical recovery resource. Those who replaced idle time with farming, running, household responsibilities, or church duties described reduced craving for substances. These narratives align with behavioural-activation theory, which emphasises engagement in positive, value-driven activities as a beneficial adjunct to traditional treatment methods [90,91].

The supportive anchors identified in this study are consistent with the Recovery Capital Framework [92], which conceptualises recovery as dependent on the internal and external resources individuals draw upon to initiate and sustain change. However, unlike some high-income settings where recovery capital is often framed around individual agency, financial resources, and professional networks, our findings suggest that recovery capital in Uganda is strongly relational and community-embedded. Faith networks, peer mentors, family engagement, and CHWs functioned as collective recovery resources rather than individually owned assets. Recovery-oriented care in Uganda therefore cannot be modelled on individual behavioural change programs alone but should deliberately mobilise and strengthen the relational and community structures that participants identified as their primary sources of recovery support.

Overall, this study contributes to policy debates on SUD care in LMICs by showing that the central challenge is not only limited treatment capacity, but also weak continuity between hospitals, families, communities, and lower-level health services. This distinction has important implications for how investment in SUD care should be prioritised and structured.

### Study limitations

While this study provides valuable insights into the multilevel barriers and facilitators shaping treatment journeys through HCWs’ and SUs’ perspectives, findings should be interpreted with acknowledgement of some limitations.

The HCW sample did not include all professional cadres involved in SUD care, such as psychiatrists, occupational therapists and social workers. As a result, the HCW findings should be interpreted as reflecting the perspectives of selected frontline providers rather than the full multidisciplinary healthcare workforce. Additionally, factors unique to these two settings may have influenced the identified themes, limiting transferability to other contexts.

Although random selection within the parent cohort minimised investigator bias, it may have limited the ability to purposively recruit participants with specific treatment trajectories or recovery profiles, such as those with sustained long-term recovery. These perspectives may therefore be underrepresented.

We did not conduct formal member-checking, which may have limited participant validation of the final themes. However, credibility was strengthened through triangulation across participant groups and sites, independent coding by two analysts, and team-based analytic review. Additionally, emerging themes and illustrative quotations were reviewed by co-authors with direct facility-based experience in SUD care to support contextual validation.

Some research assistants were clinicians involved in the parent cohort study, which may have influenced how participants discussed service quality. SUs may have been reluctant to openly criticise care if they perceived interviewers as connected to the treatment system, introducing potential social-desirability bias. In addition, HCW accounts may have reflected their specific professional roles and experiences within the health system, rather than the full range of perspectives across all cadres involved in SUD care.

## Conclusions

This study provides a dual-perspective account of how health-system constraints, fragmented care, unsupportive post-discharge environments, and individual recovery resources interact to shape SUD treatment and recovery in Uganda. Participants’ accounts suggest that the central challenge is not only limited treatment capacity, but also weak continuity between hospitals, families, communities, and lower-level health services. This weak continuity appeared to constrain translation of facility-based stabilisation into sustained recovery support beyond discharge. At the same time, participants identified locally available recovery anchors, including spiritual faith, peer mentorship, family involvement, community engagement, and active coping, that could be strengthened within a recovery-oriented system of care.

These findings emphasise the need to shift towards a continuum-of-care approach that integrates facility-based treatment with community-level interventions. Policy priorities should include expanding human resource capacity through task-sharing, embedding psychosocial and family-inclusive interventions within standard care, and ensuring reliable access to essential medications. Addressing social determinants of relapse such as poverty, stigma, and economic dependency on substance-related livelihoods will also be critical to supporting recovery beyond treatment settings.

## Supporting information

S1 Text Semi-structured Interview guides used to collect data from service users receiving treatment for substance use disorders and their healthcare workers. (DOCX)

S1 Checklist PLOS Global Public Health inclusivity in global research questionnaire. (DOCX)
