# Supplementary material for: Service user and healthcare-worker perspectives on Substance Use Disorder treatment journeys, relapse, and recovery in Sub-Saharan Africa: Evidence from Uganda
Source: PLOS Glob Public Health. 2026 Aug 21;6(8):e0005691. doi: 10.1371/journal.pgph.0005691 (PMC13496416; doi:10.1371/journal.pgph.0005691)
Supplement: Supplementary file 1 [file pgph.0005691.s001.docx]

**AQUALEFF In-Depth Qualitative Interview Guide - Service Users**

- Could you please tell me a bit about yourself; such as your age, gender, occupation, or family situation?
- When did you start receiving treatment for substance use?
- How long had you been using substances before you sought treatment?
- What substances were you using?
- What motivated you to seek treatment initially? Was there a specific event or moment that pushed you to get help?
- Can you describe your experience with the treatment program you underwent?
- What kinds of therapies, interventions, or activities did you receive during treatment?
- How did you feel when you first started treatment? Did you have any concerns, fears, or doubts at the beginning?
- After leaving the treatment program, what were the first few months like for you? What changes did you notice in yourself during that time?
- Did you face any specific challenges after completing treatment? Can you describe your substance use over the past year?
- If you relapsed, what do you think triggered it?
- How do you feel today compared to when you first completed treatment, physically, mentally, and emotionally?
- What impact has the treatment had on your daily life, relationships, work or school, physical health, or mental health?
- Do you currently receive any form of support, such as counseling, support groups, or medication? If yes: How helpful has it been? If no: What led you to stop?
- Looking back over the past year, do you feel the treatment you received was effective?
- In what ways was the treatment helpful, and in what ways was it lacking? Is there anything about the treatment program you would change or improve?
- Were there any parts of the treatment that you found particularly difficult or unhelpful?
- What aspects of the treatment did you find most beneficial in helping you stay on track?
- What challenges have you faced in staying away from substances since completing treatment?
- Have cravings, stress, social pressure, or access to substances made things difficult at times? Were there any moments when you felt especially vulnerable to relapse? If yes, what helped you get through those moments?
- How have your relationships with family, friends, or others influenced your recovery? Have they helped or hindered you?
- What coping strategies have been most effective for you in maintaining sobriety or reducing substance use?
- How do you manage stressful situations or emotional triggers without turning to substances?
- Looking ahead, what do you think will be important for your continued recovery?
- What advice would you give to someone who is just beginning their recovery journey?
- What recommendations do you have for improving substance use treatment programs, based on your own experience?
- Are there particular supports, services, or resources you feel would have made a difference for you?
- Is there anything else you would like to share about your experience that we haven’t talked about?

**AQUALEFF Qualitative Interview Guide – Health Care Workers**

- Can you tell me about your role in this facility and your experience working with patients who have substance use disorders?
- Walk me through what typically happens when a patient with substance use problems first arrives at this facility.
- How do you assess and determine whether a patient should be admitted to the SUD unit?
- What tools or approaches do you use to screen, assess, or diagnose patients with substance use problems?
- How do you identify co-occurring mental or physical health conditions in these patients?
- Can you describe the treatment approaches used here for managing substance use disorders?
- How do you incorporate biological, psychological, or social components of care in your daily work?
- How do you typically manage withdrawal or detoxification for different substances?
- What challenges arise during detoxification or acute management?
- What has been your experience with the availability of essential medications and supplies needed for SUD treatment?
- How do you handle situations where key medicines or resources are unavailable?
- What forms of counseling, group work, occupational therapy, or family involvement are available for patients?
- In your experience, how do these psychosocial supports affect patient recovery?
- What is it like working in terms of staffing, workload, and available expertise on the unit?
- How do staffing levels affect the quality or consistency of patient care?
- From your perspective, what are the most common reasons patients relapse after discharge?
- What factors help patients maintain sobriety or recovery?
- How is discharge usually planned and implemented for SUD patients?
- What kind of follow-up or community support exists after patients leave the facility?
- What facility-level or system-level challenges affect the delivery of SUD treatment here?
- How do issues like stigma, infrastructure, or security influence your work with patients?
- What changes or improvements do you think could strengthen SUD treatment and recovery support in this facility?
- What community- or system-level strategies do you believe would improve long-term recovery outcomes?
- Is there anything else about your experience working with people with substance use disorders that you think is important for us to understand?
