# Supplementary material for: Service user and healthcare-worker perspectives on Substance Use Disorder treatment journeys, relapse, and recovery in Sub-Saharan Africa: Evidence from Uganda
Source: PLOS Glob Public Health. 2026 Aug 21;6(8):e0005691. doi: 10.1371/journal.pgph.0005691 (PMC13496416; doi:10.1371/journal.pgph.0005691)
Supplement: Supplementary file 2 [file pgph.0005691.s002.docx]

Inclusivity in global research

PLOS’ policy on inclusivity in global research aims to improve transparency in the reporting of research performed outside of researchers’ own country or community and ensures that PLOS publications reporting global research adhere to high standards for research ethics and authorship. Authors of relevant research articles may be asked to complete the questionnaire below, which outlines ethical, cultural, and scientific considerations specific to inclusivity in global research. This questionnaire may be requested when researchers have travelled to a different country to conduct research, if research uses samples collected in another country, research with Indigenous populations or their lands, or if research is on cultural artefacts. Researchers travelling to another country solely to use laboratory equipment will not normally be required to complete the questionnaire. However, the questionnaire can be requested at the journal’s discretion for any submission – if you have been requested to complete this questionnaire by the PLOS journal you submitted to, please do so.

Please complete the questionnaire below and include this as a Supporting Information file with your manuscript. Note that if your paper is accepted for publication, this checklist will be published with your article in the supporting information files. Please ensure that you reference the checklist in the main body of your manuscript. We suggest adding a subsection ‘Inclusivity in global research’ to your Methods section and adding the following sentence: “Additional information regarding the ethical, cultural, and scientific considerations specific to inclusivity in global research is included in the Supporting Information (SX Checklist)”

The questions have been designed to be applicable to a wide range of study types, and there are subsections for both human subjects research and non-human subjects research. If any of the questions are not relevant to your research please mark them as “N/A” as appropriate.

**Ethical considerations, permits and authorship**

*This section is applicable to all research types.*

Provide details as to who granted permissions and/or consent for the study to take place in the Methods section of your manuscript. This should include the names of **all** ethics boards, governmental organizations, community leaders or other bodies that provided approval for the study. If individuals provided approval refer to these people by their role or title but do not list their name(s).

Reported on page number: 5 - 6
Ethical approval was obtained from Makerere University School of Public Health Research Ethics Committee (MakSPH-REC) as part of the AQUALEFF study (Advancing Quality and Effectiveness of Treatment of Substance Use Disorders in Uganda). The study was subsequently registered with Uganda National Council for Science and Technology in accordance with national research guidelines under Registration Number HS6699ES. Institutional administrative clearance to conduct research at the treatment facilities was obtained from the treatment facilities where data collection took place.

If there were any deviations from the study protocol after approval was obtained please provide details of these changes in the Methods section of your manuscript.

Reported on page number: NA
No deviations from the approved study protocol were made following receipt of ethical approval. The study was conducted in accordance with the approved protocol throughout the data collection period.

Did this study involve local collaborators that are residents of the country where the research was conducted or members of the community studied? If you do not have any authors from said communities, please provide an explanation for this below.

Yes
This study was conceptualised, led, and conducted by Ugandan researchers based in Uganda. The first author (Claire Biribawa) is a Ugandan PhD candidate based at Makerere University School of Public Health (MakSPH), Kampala, Uganda. The primary Ugandan supervisor (Prof. Nazarius Mbona Tumwesigye) is a senior Ugandan researcher at MakSPH with extensive experience in alcohol and substance use research in Uganda. All data collection was conducted by the first author and Ugandan research assistants fluent in the relevant local languages.

International co-authors (Prof. Wouter Vanderplasschen and Prof. Vicki Simpson) contributed to study design, analytical supervision, and manuscript development. All authors meet PLOS criteria for authorship and are listed in the author byline accordingly.

Everyone listed as an author should meet PLOS’ criteria for authorship and all individuals who meet these criteria should be included in the author byline, rather than the acknowledgements. For further information please see the journal’s Authorship Policy.

**Human subjects research (e.g. health research, medical research, cross-cultural psychology)**

Did you obtain written informed consent from a representative of the local community or region before the research took place? How did you establish who speaks for the community? Details of written informed consent obtained from study participants should be reported separately in the Methods section of your manuscript.

How did members of the local community provide input on the aims of the research investigation, its methodology, and its anticipated outcome(s)?

The study design was informed by established clinical and research relationships between the research team and the two participating facilities, developed through prior research collaborations and the parent cohort study from which service user participants were drawn. Administrative clearance from both facility managements was sought prior to commencing data collection, and facility-level contacts provided input on feasibility, appropriate sampling procedures, and logistical arrangements.

Importantly, the research assistants who conducted service user interviews were trained healthcare workers employed at BNRMH and GRRH respectively. Their involvement went beyond data collection to frontline practitioners with direct knowledge of the patient population, treatment processes, and local context, who provided substantive input into the refinement of interview guides and data collection procedures during the training and piloting phase..

When engaging with the local community, how did you ensure that the informed consent documents and other materials could be understood by local stakeholders?

All data collection tools, including informed consent forms, and interview guides, were translated from English into the relevant local languages Luganda, Acholi, and Swahili by bilingual research assistants fluent in both English and the respective local language.

Research assistants were trained to explain the study aims, procedures, voluntary nature of participation, and confidentiality provisions verbally in participants' preferred languages prior to obtaining written consent. To confirm understanding, participants were asked comprehension check questions after the informed consent information was provided and before signing, ensuring they had genuinely understood what their participation entailed. Participants had the opportunity to ask questions at any point before and during the interview. Interviews were conducted in participants' preferred languages to ensure natural and accessible communication throughout data collection.

Will the findings of the research be made available in an understandable format to stakeholders in the community where the study was conducted (e.g. via a presentation, summary report, copies of publications, etc.)? Please provide details of how this will be achieved.

Yes.

A summary report of key findings will be shared with stakeholders at each participating facility including planned dissemination activities to clinical and management staff at BNRMH and GRRH, a briefing to relevant officials at the Uganda Ministry of Health, particularly those responsible for mental health and substance use disorder policy; dissemination at national and international conferences; and open-access publication, ensuring the findings are freely accessible to Ugandan researchers, clinicians, and policymakers without financial barriers.

**Non-human subjects research using specimens/ animals collected as part of the study, or those housed in archival collections. Examples include archaeology, paleontology, botany and zoology.**

Did the permission you obtained from a local authority to perform the study include an agreement on access to outputs and benefit sharing? This may include procedures to enable fair distribution of the benefits and resources arising from the research performed. Please include any details of Prior Informed Consent and Benefit Sharing Agreements obtained. These may be required by field-specific regulations, for example the Convention on Biological Diversity (CBD) and the associated Nagoya Protocol.

Not Applicable

If the material used in your study was imported, please A) provide the year it was imported and B) indicate whether permits were obtained to import/export the materials used, C) provide details of any permits obtained. If this information is not available, please indicate this.

Not Applicable

If you used archival specimens, please state how the material used in your study was acquired by the institute it is held in and provide details of any permits obtained for the original excavations/ sample collection. If this information is not available, please indicate this.

Not Applicable

How was the potential cultural significance of the materials collected in your study to local communities considered in your research design? Were Indigenous peoples and/or local researchers and institutions involved with archaeological excavations / collection of specimens? If so, please provide a description of their involvement.

Not Applicable

If your manuscript includes photographs of human remains please indicate whether authors obtained permission from descendants or affiliated cultural communities to do so.

Not Applicable
